# Role of VPAC1 and VPAC2 receptors in the etiology of pregnancy rhinitis: an experimental study in rats

**DOI:** 10.1016/j.bjorl.2020.06.015

**Published:** 2020-08-01

**Authors:** Burak Ulkumen, Muhammet Burak Batir, Burcu Artunc Ulkumen, Halil Gursoy Pala, Seda Vatansever, Sirri Cam

**Affiliations:** a Manisa Celal Bayar University, Faculty of Medicine, Department of Otorhinolaryngology-Head Neck Surgery, Manisa, Turkey; b Manisa Celal Bayar University, Faculty of Science and Letters, Department of Biology, Manisa, Turkey; c Manisa Celal Bayar University, Faculty of Medicine, Department of Obstetrics and Gynecology, Manisa, Turkey; d The University of Health Sciences Tepecik Training and Research Hospital, Department of Obstetrics and Gynecology, İzmir, Turkey; e Manisa Celal Bayar University, Faculty of Medicine, Department of Histology-Embryology, Manisa, Turkey; f Near East University, Experimental Research Center of Health (DESAM), Mersin, Turkey; g Manisa Celal Bayar University, Faculty of Medicine, Departamento de Genética Médica, Manisa, Turkey

**Keywords:** Pregnancy rhinitis, VPAC1, VPAC2, Estradiol, Progesterone

## Abstract

**Introduction:**

Pregnancy rhinitis is a common sex hormone-related otorhinolaryngological disorder. There are some epidemiological and physiological studies on pregnancy rhinitis, but histopathological and biomolecular changes have not been studied thoroughly.

**Objectives:**

The receptors VPAC1 and VPAC2 are known for their roles in allergic rhinitis. On the other hand, activation of subclinical allergy has been suggested in the pathophysiology of pregnancy rhinitis. Therefore, we aimed to compare the physiological and gestational pattern of VPAC1 and VPAC2 expression in rat nasal mucosa.

**Methods:**

Twenty adult Wister albino female rats were enrolled into the study. Two groups constituted as 10 control (group A) and 10 pregnant (group B) rats. They were fed ad libitum and sheltered at room temperature (22°±2 °C). The rats were sacrificed at the 20th day of gestation by intraperitoneal injection of 400 mg/kg Na-pentobarbitone. Then, 10 − 15 mL of blood was taken, and samples were reserved for the detection of serum estradiol and progesterone levels by ELISA test. The nasal septum was resected and divided in half for immunohistochemical analyses and real time polymerase chain reaction testing of VPAC1 and VPAC2.

**Results:**

VPAC1 and VPAC2 were found to be in all layers of septal specimens, but the immunostaining of surface epithelium was more distinct in specimens of both groups. We demonstrated higher overall staining intensity in the pregnant group. PCR revealed significant increase in expression of VPAC1 (p = 0.023) and VPAC2 (p = 0.021) in pregnant group when compared with control group. In addition, we demonstrated upregulatory effect of estradiol and progesterone on the vasoactive intestinal peptide receptor expression.

**Conclusions:**

Gestational up-regulation of nasal VPAC1 and VPAC2 was shown both by PCR and immunohistochemical analysis. These findings support the hypothesis that PR is caused by the activation of subclinical allergy that is present before pregnancy.

## Introduction

Fluctuations in serum gonadocorticoid levels cause some adverse Otorhinolaryngological (ORL) signs and symptoms.^1^, ^2^ It is known that physiological, pathological or iatrogenic conditions cause change in gonadocorticoid levels.^3^, ^4^, ^5^ Pregnancy, as a physiological condition, also induces some sex hormone- related ORL symptoms.^6^, ^7^ One of them is Pregnancy Rhinitis (PR), which is defined as the presence of nasal congestion, rhinorrhea and sneezing which arise particularly during gestation and subside in 3 weeks’ time after delivery. In addition, history of allergy and other nasal pathologies are defined as exclusion criteria for PR.^3^, ^8^

PR leads to a predisposition to snoring and even obstructive sleep apnea syndrome, which induces some maternal and fetal morbidities.^9^, ^10^, ^11^ The pathophysiology of PR is still not fully understood. But increased serum levels of particular hormones (Progesterone [PG], Estradiole [E2], placental growth hormone, human chorionic gonadotropin) have been asserted as the triggering factor.^10^ Activation of subclinical allergy has also been suggested as the pathophysiology of PR by some studies.^3^, ^12^, ^13^, ^14^ However, there is no high level of evidence to support that Allergic Rhinitis (AR) and PR share similar pathophysiological processes. In addition, the impact of sex hormones on nasal mucosa has been studied in various aspects.^15^, ^16^, ^17^, ^18^, ^19^, ^20^ But there is very limited data regarding histopathological and biomolecular changes in the nasal mucosa of patients having PR.^21^, ^22^

Vasoactive Intestinal Peptide (VIP) is a peptide hormone which is known to be secreted in various tissues.^23^ VIP is mainly known for its immunomodulatory functions.^24^ It takes part in regulation of immune response through 4 receptors (VPAC-1, VPAC-2, PAC1, CRTH2). In the upper respiratory tract, VIP mainly functions via VPAC1 and VPAC2. On the other hand, upregulation of nasal VPAC-1 and VPAC-2 expression was reported in AR.^25^ The link between these two receptor proteins and allergy was also shown in some other studies.^24^, ^26^ In consideration of these findings we hypothesize that the pattern of VPAC-1 and VPAC-2 expression would significantly change in the nasal mucosa of pregnant rats when compared with non-pregnant ones. Discovery of any gestational change in nasal expression of these 2 biomolecules may partly unveil the histopathological background of PR. Besides, we may have the opportunity to reveal a common pathway which would link up the physiopathology of AR and PR. By this means new treatment strategies could be proposed for PR.

As far as we know, the nasal expression of VPAC1 and VPAC2 has not been studied in relation to pregnancy before. In this study, we explored the physiological pattern of VPAC-1 and VPAC-2 expression in rat nasal mucosa as well as the impact of pregnancy on these receptors. Additionally, the effect of PG and E2 on the expression of these 2 receptors was also studied.

## Methods

### Animals

This animal experimental study was done in institutional experimental animals’ research and application center in accordance with the accepted policy on the use of animals. Institutional Animal Ethics Committee approved the overall procedures.

Twenty adults (8 − 12 week old) Wister albino female rats were involved in the study. The rats were fed ad libitum and sheltered at room temperature (22°±2 °C) on a 12 h light-dark cycle. All procedures were done during daytime. Thirty-two female rats were housed together with male rats in the ratio of 4/1 for overnight. The following morning vaginal smears of female rats were examined for sperm existence. We accept the observation of sperm as the day 0 of the gestation as described before.^27^, ^28^, ^29^ The first 10 rats with negative and 10 rats with positive vaginal smear were chosen from among 32 rats as the control group (Group A) and the pregnant group (Group B), respectively. The remaining 12 rats were not enrolled into the study. Gestation period of Wister albino rat has been reported as approximately 22 (21 − 26) days.^29^ For this reason, we sacrificed the rats at 20thday of gestation by intraperitoneal injection of 400 mg/kg Na-pentobarbiton.^30^ After the loss of consciousness and righting reflex, 10 − 15 mL of blood was taken by a 23 G needle.. Blood samples were reserved for the detection of serum E2 and PG levels by ELISA.

**Figure 1:**
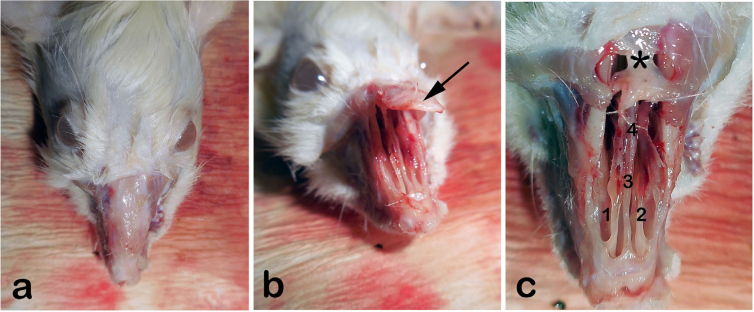
(a) External view of rat’s rhinarium (dorsal hair shaved), (b) Nasal roof (Os nazale) are mobilized in upward direction, (c) Internal macroscopic view of both nasal cavities (Cavum nasi) after removal of nasal roof. The septum (Septum nasi osseum), middle nasal chonca (Concha nasalis media) and frontal bone (Os frontale) are seen. Arrow: Os nazale; 1 and 2: Middle nasal conchae; 3: Septum with respiratory epithelium; 4: Vomer with olfactory mucosa; * Frontal bone.

We then shaved the nasal dorsum (Fig. 1a). We separated the nasal bones from the maxilla in an upward manner and revealed the nasal cavity macroscopically as described by Alvites et al. (Fig. 1b).^30^ We then resected the cartilaginous part of the septum, taking care not to damage the mucosal integrity. The resected specimen was divided in half for immunohistochemical analyses and real- time Polymerase Chain Reaction testing (PCR).

### Immunohistochemistry (IHC)

The pecimens were fixed by formaldehyde solution of 10% for 24 h. Next, dehydration and paraffin emplacement were accomplished. Paraffin blocks were dissected at a 4 μm thickness. For primary examination, Hematoxylin-Eosin (H&E) dye was utilized. “VPAC1 Antibody (B-4): sc-377152” and “VPAC2 Antibody (AS69): sc-52795” were used in accordance with the recommended protocols for immunohistochemical detection of VPAC1 and VPAC2 (Santa Cruz Biotechnology, Inc., TX, USA). Concentration of VPAC1 and VPAC2 was 200 μg/mL and 50 μg/mL, respectively. The control of immunospecifity of each group was performed by the replacement of primary antibodies with Phosphate Buffered Saline (PBS). Immunohistochemical pattern of VPAC1 and VPAC2 expression was examined by light microscopy (Olympus BX41). Immunostaining for VPAC1 and VPAC2 was evaluated in terms of structural layers of nasal mucosa.

### ELISA

EDTA, sodium citrate and heparin were mixed with the blood samples. Then the mixture was centrifuged at 3000 rpm for 10 min. The supernatant stored at −80 °C. Rat E2 (estradiol) ELISA Kit and General Progesterone (PG) ELISA Kit were used for quantitative measurement of serum E2 and PG levels, respectively (MyBioSource, Inc., CA, USA).^29^

### RNA extraction and quantitative Real-Time PCR (qRT-PCR) analyses

Total RNA extraction was carried out from the laryngeal mucosa with using TRIzol® Reagent in the combination with the PureLink® RNA Mini Kit (Thermo Fisher Scientific, 12183555). RNA expression levels of VPCA1 and VPCA2 were acquired from the extracted RNA samples with using forward VPCA1F1 primer 5′-ATCAACTCCTCCCTGTGGTG-3′, reverse VPCA1R1 primer 5′-GGGCTGCTATCATTCTTCCC-3′, forward VPCA2F1 5′-GGACAGTGTGCTCTACTCCA-3′, reverse VPCA2R1 5′-GCCAGTAGAAGTTCGCCATG-3′ and QuantiFast SYBR Green qRT-PCR Kit (Qiagen, 204154). Separately prepared mixture of AQP5F1, AQP5R1, QuantiFast SYBR Green and TREKF1, TREKR1, QuantiFast SYBR Green was analyzed in the Rotor-Gene Q (Qiagen, Hilden, Germany). Normalising of the target genes expression changes was performed according to the β-microtubulin (B2M) (B2MF1 5′-TCTCTCTTTCTGGCCTGGA-3′, B2MR1 5′-TGTCGGATGGATGAAACCC-3′) and Hypoxanthine Phosphoribosyl Transferase (HPRT1) (HPRT1F1 5′-CGTCTTGCTCGAGATGTGAT-3′, HPRT1R1 5′-TTCAGTGCTTTGATGTAATCCAG-3′) housekeeping gene expression values. The related forward and reverse primers were synthesized by Metabion company (Germany). qRT-PCR cycling conditions started with the reverse transcription step of 50 °C (10 min), followed by PCR step comprised of an initial activation/denaturation stage of 95 °C (5 min), followed by 45 cycles of denaturation 95 °C (20 s), combined annealing/extension 61 °C (50 s). The 2^−ΔΔCT^ method was used to calculate the relative changes in gene expression.^31^

### Statistical analyses

Relative VPAC1 and VPAC2 expressions were compared between Group A and B. Distribution of data were evaluated by Shapiro-Wilk test and results were presented as mean ± Standard Deviation (SD). Variables of Group A and B were compared by independent samples *t*-test or Mann-Whitney *U* Test according to the results of Shapiro-Wilk test. The impact of serum E2 and PG levels on VPAC1 and VPAC2 was analyzed by Pearson correlation test. Statistical significance was defined as p < 0.05. The Statistical Package for the Social Sciences (SPSS) Version 21.0 (IBM Corp.; Armonk, NY, USA) was used for statistical calculations.

## Results

### Immunohistochemistry

**Figure 2:**
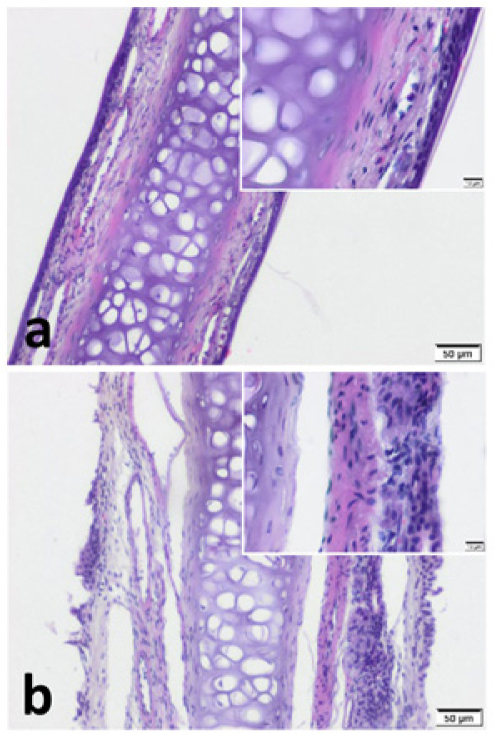
Septal cartilage with covering mucoperichondrium. Increased cellularity and sub-epithelial oedema is notable in pregnant group. (a) Control group (b) Pregnant group (H&E stain) (Scale Bars = 50 μm and 10 μm).

We observed pseudostratified cuboidal-columnar type of epithelium in Hematoxylin and Eosin (H&E) sections of nasal septal cartilage. In addition, increased superficial cellularity and sub-epithelial edema was notable in Group B when compared with the Group A (Fig. 2).

**Figure 3:**
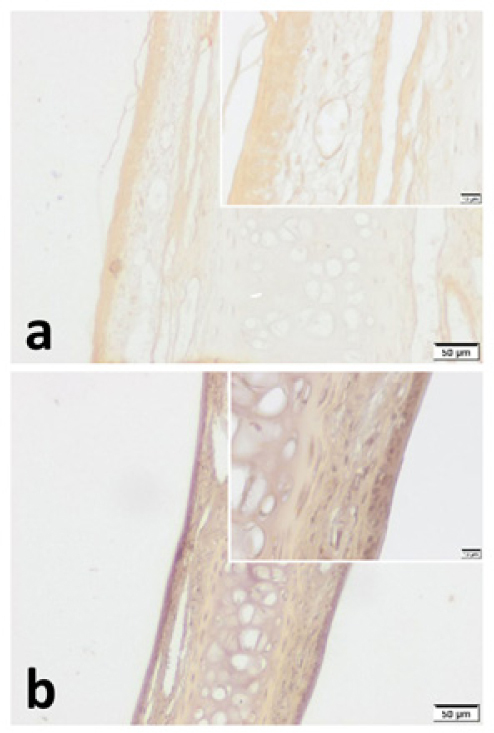
VPAC1 immunohistochemistry for (a) Control group and (b) Pregnant group. VPAC1 was found to be expressed in all layers of septal mucoperichondrium. Additionally, it was more intense in the surface epithelium of both groups. Overall staining intensity was more explicit in specimens of pregnant group. (Scale Bars =50 μm and 10 μm).

**Figure 4:**
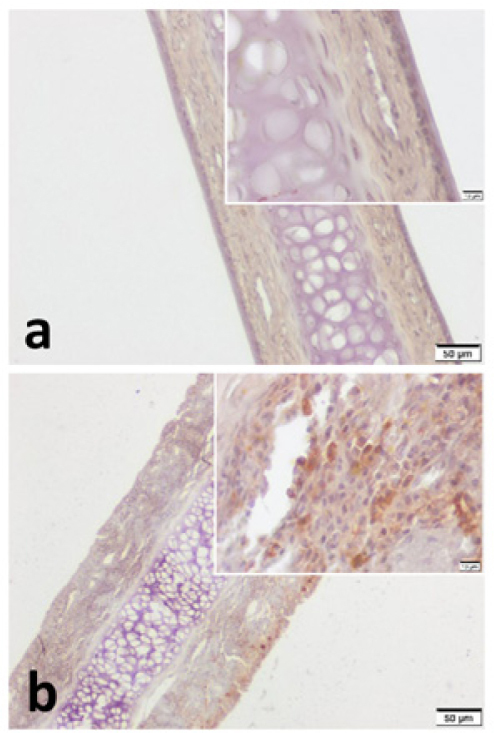
VPAC2 immunohistochemistry for (a) Control group and (b) Pregnant group. VPAC2 was found to be stained in all layers of septal mucoperichondrium. Subepithelial hypercellularity was remarkable in the pregnant group (b). Overall staining intensity was more explicit in specimens of pregnant group. (Scale Bars =50 μm and 10 μm).

VPAC1 and VPAC2 were found to be located in all layers of septal specimen (epithelium, basement membrane, lamina propria, cartilage). But the staining intensity of epithelium was more distinct in specimens of both group (Figure 3, Figure 4). Although we did not use an objective measure like “H-Scoring” for comparison of groups, we roughly observed higher overall staining intensity in samples of Group B when compared with Group A (Fig. 3b, Fig. 4b).

### PCR and ELISA

A total of 20 Wister albino female rats (10 control, 10 pregnant) were enrolled into the experiment. The mean relative expression of mRNA coding for VPAC1 of Group A and B was 0.029 ± 0.016 and 0.055 ± 0.026, respectively. The mean relative expression of mRNA coding for VPAC2 of Group A and B was 0.003 ± 0.001 and 0.007 ± 0.004, respectively. The mean serum E2 levels of Group A and B was 21.85 ± 1.45 pg/mL and 73.59 ± 3.01 pg/mL, respectively. The mean PG levels of Group A and B was 14.99 ± 1.96 ng/mL and 32.80 ± 3.96 ng/mL, respectively (Table 1). PCR and ELISA values were found to be abnormally distributed (p < 0.05). For this reason, Mann-Whitney *U* Test was used for comparison of relative biomolecule expressions and serum sex hormone (E2, PG) levels between groups.

**Table 1:** Expression of VPAC1 and VPAC2 in nasal mucosa of rat, serum E2 and PG levels based on groups.

Biomolecules & sex hormones	Control (Group A)	Pregnant (Group B)	p-value^a^
Biomolecules			
VPAC1 (REV)	0.029 ± 0.016	0.055 ± 0.026	p = 0.023
VPAC2 (REV)	0.003 ± 0.001	0.007 ± 0.004	p = 0.021
Serum sex hormone levels			
Estradiol (pg/mL)	21.85 ± 1.45pg	73.59 ± 3.01 pg/mL	p<0.001
Progesterone (ng/mL)	14,99 ± 1.96 ng/mL	32.80 ± 3.96 ng/mL	p<0.001

REV, Relative expression value.

a *p*-values obtained by Mann-Whitney U Test.

Both relative VPAC1 (*p* =  0.023) and VPAC2 (*p* =  0.021) expression was found to be significantly higher in Group B when compared with Group A. E2 and PG were also found significantly higher in Group B when compared with Group A (*p* <  0.001) (Table 1).

## Discussion

PR is a relatively common sex hormone-related ORL disorder. Its incidence has been reported between 10%−40%.^8^, ^12^ Unfortunately, the awareness of PR among patients and even physicians is quite low. PR may cause some serious comorbidities like maternal hypertension, preeclampsia, low APGAR score and fetal growth retardation.^10^, ^11^, ^32^ There are some epidemiological and physiological studies on PR,^8^, ^33^, ^34^ but histopathological and biomolecular background has not been studied thoroughly. Besides, there are very limited numbers of studies regarding the treatment for this quite common disorder.^12^, ^32^, ^34^, ^35^ Suggestions for any treatment modality necessitates comprehension of physiopathology and histopathology. For this reason, in this animal experimental study, we aimed to reveal histochemical and biomolecular (VPAC1 and VPAC2) changes of nasal mucosa during gestation.

VIP plays a key role in allergic diseases like AR and asthma. It particularly functions via VPAC1 and VPAC2 in the upper respiratory tract.^23^ Stimulation of these 2 receptors leads secretion of pro- and anti-inflammatory mediators like prostaglandins and leukotrienes. This ends up with extravasation of inflammatory cells into the mucosa. Other well-known functions of VIP are bronchodilation, vasodilation and increased glandular secretion. In addition, recent studies emphasize the neuroimmune interaction of VIP in the context of allergic diseases.^23^, ^36^ All these effects may also play a role in the etiopathogenesis of PR. In fact, some researchers have been asserted that PR is caused by aggravation of preexisting subclinical allergy.^3^, ^12^, ^13^ Toppozada et al. revealed some changes like AR in specimens of PR patients by electron microscopy.^13^ On the other hand, Ellegard et al. revealed increased levels of IgE against house dust mite in PR patients.^14^ In this study, we revealed a statistically significant increase of nasal VPAC1 (*p* =  0.023) and VPAC2 (*p* =  0.021) expression in pregnant rats when compared with the non-pregnant ones (Table 1). This finding supports the argument of previous researchers.^13^, ^14^ In other words, this study supports the hypothesis that PR and AR may share a common pathway for VIP.

Impact of sex hormones on the nasal mucosa has been shown by previous researchers in various physiological, iatrogenic or pathological conditions.^3^, ^5^, ^6^, ^7^, ^13^, ^15^, ^16^, ^17^, ^18^, ^19^, ^20^, ^21^, ^22^ Almost all concluded that E2 and PG causes obstruction and edema in the nasal cavity which is the case in this study (Fig. 2b).^3^, ^6^, ^13^, ^15^^,^^16^, ^21^ In addition to these findings, we found a relatively increased superficial higher cellularity in the epithelial layer in samples of Group B. This may be a sign of increased metabolism or activity. On the other hand, very few researchers evaluate the underlying histochemical and biomolecular changes. Konno et al. revealed that E2 up-regulates cholinergic muscarinic receptors while PG down-regulates α_1_-adrenergic receptors.^20^ These findings of Konno et al. are compatible with the study of Fisher et al. in relation to neuroimmunomodulation.^36^ Fisher et al. revealed higher VIP contents in mucosal nerve fibers of AR patients when compared with control. Our findings also support this phenomenon. The higher nasal VPAC1 and VPAC2 expression in pregnancy that was revealed in this study can also be the triggering factor for PR. Besides, Philpott et al. discovered a positive correlation between oestrogen-β (ERβ) receptor expression and rhinitis symptoms.^17^ Our findings also support this correlation. We revealed up-regulatory effect of E2 and PG on VPAC1 by PCR. But VPAC2 was found to be stimulated only by E2. These findings may be the missing link of the VIP pathway triggered by sex hormones, resulting in extravasation of inflammatory cells into the nasal mucosa.

In the current study, both VPAC1 and VPAC2 were found to be located in all layers of septal mucoperichondrium. However, the surface epithelium of both groups demonstrated higher immunostaining for both VIP receptors. In addition, we revealed higher overall staining intensity in samples of pregnant rats (Figure 3, Figure 4). These IHC findings are similar to the study of Kim et al.^25^ They demonstrated increased staining intensity for VIP receptors in nasal mucosa of AR patients. Therefore, we can suggest that PR and AR have a similar effect on VIP receptors. On the other hand, this strong immunostaining particularly demonstrated in the surface epithelium can be interpreted as a sign of hyper responsiveness of nasal mucosa in pregnancy.

## Conclusions

We revealed gestational upregulation of VPAC1 and VPAC2 expression in rat nasal mucosa for the first time both by PCR and IHC. The current study supports the hypothesis that PR is caused by the activation of a subclinical allergy that is present before pregnancy. Further studies are needed to unveil the complete histopathological and biomolecular changes regarding PR.

## Ethical approval

This animal experimental research was approved by the Laboratory Animals Local Ethics Committee of Manisa Celal Bayar University (28.04.2015/77.637.435-29) and was performed in institutional Experimental Animals Research and Application Center in accordance with the accepted policy on the use of animals.

Committee Name: The Laboratory Animals Local Ethics Committee of Manisa Celal Bayar University. Permit number: (28.04.2015/77.637.435-29).

## Conflicts of interest

The authors declare no conflicts of interest.

## Funding

Unit of Scientific Research Projects (10.13039/501100000544BAP) of Manisa Celal Bayar University (Grant number: 2014-131).
